# Minimally invasive colorectal surgery in the morbid obese: does size really matter?

**DOI:** 10.1007/s00464-018-6068-5

**Published:** 2018-01-23

**Authors:** Sofoklis Panteleimonitis, Sotirios Popeskou, Mick Harper, Ngianga Kandala, Nuno Figueiredo, Tahseen Qureshi, Amjad Parvaiz

**Affiliations:** 1 School of Health Sciences and Social Work, University of Portsmouth, James Watson West, 2 King Richard 1st road, Portsmouth, PO1 2FR UK; 2 Poole Hospital NHS Trust, Longfleet road, Poole, BH15 2JB UK; 3 Champalimaud Foundation, Av. Brasilia, 1400-038 Lisbon, Portugal; 4 Bournemouth University School of Health and Social Care, Bournemouth, UK

**Keywords:** Minimally invasive, Laparoscopic, Colorectal surgery, Obese

## Abstract

**Background:**

As obesity becomes more prevalent, it presents a technical challenge for minimally invasive colorectal resection surgery. Various studies have examined the clinical outcomes of obese surgical patients. However, morbidly obese patients (BMI ≥ 35) are becoming increasingly more common. This study aims to investigate the short-term surgical outcomes of morbidly obese patients undergoing minimal-invasive colorectal surgery and compare them with both obese (30 ≤ BMI < 35) and non-obese patients (BMI < 30).

**Methods:**

Patients from three centres who received minimally invasive colorectal surgical resections between 2006 and 2016 were identified from prospectively collected databases. The baseline characteristics and surgical outcomes of morbidly obese, obese and non-obese patients were analysed.

**Results:**

A total of 1386 patients were identified, 84 (6%) morbidly obese, 246 (18%) obese and 1056 (76%) non-obese. Patients’ baseline characteristics were similar for age, operating surgeon, surgical approach but differed in terms of ASA grade and gender. There was no difference in conversion rate, length of stay, anastomotic leak rate and 30-day readmission, reoperation and mortality rates. Operation time and blood loss were different across the 3 groups (morbidly obese vs obese vs non-obese: 185 vs 188 vs 170 min, *p* = 0.000; 20 vs 20 vs 10 ml, *p* = 0.003). In patients with malignant disease there was no difference in lymph node yield or R0 clearance. Univariate and multivariate linear regression analysis showed that for every one-unit increase in BMI operative time increases by roughly 2 min (univariate 2.243, 95% CI 1.524–2.962; multivariate 2.295; 95% CI 1.554–3.036). Univariate and multivariate binary logistic regression analyses showed that BMI does not affect conversion or morbidity and mortality.

**Conclusions:**

The increased technical difficulty encountered in obese and morbidly obese patients in minimally invasive colorectal surgery results in higher operative times and blood loss, although this is not clinically significant. However, conversion rate and post-operative short-term outcomes are similar between morbidly obese, obese and non-obese patients.

Minimally invasive surgery (MIS) has become the new standard for colorectal diseases in the developed world, and its benefits such as shorter hospital stay, less post-operative pain, early mobilisation and improved cosmesis are well established [1–6]. These benefits are crucial for high-risk groups of patients such as the clinically obese who’s presentation is clearly linked with several comorbidities such as diabetes mellitus and cardiovascular disease in addition to higher risks of suffering from surgical site infections and pulmonary embolisms [7]. Based on this, it is speculated that a less invasive approach to surgery would be optimal for this group of patients [8].

Although obese patients have much to gain from a minimally invasive approach to colorectal surgery, the increased amount of visceral fat encountered in obese patients increases the technical difficulty of surgery [8]. Obese patients often have a thickened and excessive omentum and mesentery which restricts access, distorts the surgical planes and can result in problematic bleeding [9]. Whether the increased technical difficulty encountered in this group of patients leads to higher conversion rates and worse short-term outcomes is a subject of debate. For example, studies have examined the short-term outcomes of obese patients in laparoscopic colorectal surgery with many reporting inferior short-term outcomes in the obese [10–12] while others demonstrating similar outcomes between obese and non-obese patients [13, 14].

An ageing population, austerity and lifestyle choices are causing a rise in obesity causing health and care challenges as well as high costs to the economy. In the US more than one-third of the population is reported to be obese and in the UK obesity prevalence has risen from 15% in 1993 to 26% in 2014 [15, 16]. In addition to this, morbidly obese patients are becoming increasingly more common. According to an English report by the Health and Social Care Information Centre (2016) on the Statistics on Obesity, Physical Activity and Diet [16], in England in 2014 2% of men and 4% of women had a BMI of 40 or higher. This number has tripled over the last 25 years and presents a worrying picture, but in comparison, the USA reports 5% of the population is believed to be morbidly obese [15]. Therefore, the technical challenges discussed above for obese patients are magnified when considering minimally invasive surgery on morbidly obese patients where the risks encountered in this group of patients are even greater, due to the ever-greater amount of visceral fat encountered in the abdomen further restricting the space for manoeuvre during surgery and making it hard to define the surgical planes. Despite this, the surgical outcomes of this group of patients are poorly examined as most studies compare the outcomes of obese (BMI ≥ 30) vs non-obese patients (BMI < 30).

The study reported here aims to investigate the short-term surgical outcomes of morbidly obese (BMI ≥ 35) patients undergoing minimally invasive colorectal resection surgery and compare them to those of obese (30 ≤ BMI < 35) and non-obese (BMI < 30) patients. This study presents the biggest European series examining morbidly obese patients receiving minimally invasive colorectal resection surgery.

## Materials and methods

Consecutive patients from three centres, two from the UK and one from Portugal, who received minimally invasive colorectal surgical resections between 2006 and 2016 were identified from prospectively collected databases. All patients whose BMI was reported were included in the study. Patients were categorised as morbidly obese (BMI ≥ 35), obese (30 ≤ BMI < 35) and non-obese (BMI < 30) in accordance with the NIH conference [17].

Patients were included in the study irrespective of indication for surgery and obesity or morbid obesity was not considered a contraindication for minimally invasive surgery. All cancer patients were discussed in the multidisciplinary team meeting. Surgery was laparoscopic or robotic, with the robotic approach being preferred for all rectal surgery since the acquisition of the robot in each unit. Applied surgical modality was based on surgeon preference and equipment availability. Informed consent was obtained from all patients prior to inclusion to this study. The requirements for anonymization of personal dataset by the Data Protection Act 1998 were satisfied. According to the Health Research Authority (HRA), this study was not classified to need their approval as it is an audit.

Patients included in the study had surgery performed by three colorectal surgeons, one surgeon in each centre. Data collection began when the surgeons participating in this study started working in their respective units, between 2006 and 2012 for the UK centres and 2013 for the Portuguese centre. All surgeons applied a modular, standardised previously described approach to surgery [18–20].

Post-operative care was standardised, with patients entering a routine enhanced recovery programme based on the one described by Kehlet and Wilmore [21]. Patients were discharged home when their condition was assessed as meeting set criteria for discharge.

### Data collection and outcome assessment

All data were collected from prospectively collated databases. The baseline characteristics and surgical outcomes of morbidly obese, obese and non-obese patients were analysed. Baseline characteristics analysed were age, gender, ASA grade, diagnosis (malignant vs benign), mode (elective vs emergency), surgical approach (laparoscopic vs robotic), operating surgeon, operation performed and T stage (for malignant disease). Perioperative data included operative time, estimated blood loss and conversion to open (defined as any incision needed to either mobilise the colon or rectum or ligate the vessels). Post-operative clinical data examined included length of stay, 30-day readmission, 30-day reoperation, 30-day mortality and anastomotic leak. For malignant disease cases lymph node yield and circumferential resection margin (CRM) clearance were examined. Two subgroup analyses were performed. Morbidly obese patients with a BMI ≥ 40 were compared with patients whose BMI was ≥ 35 and < 40. Moreover, subgroup analyses of patients receiving right colonic, left colonic and rectal resections was performed.

### Statistical analysis

Data were analysed using IBM SPSS version 22 (SPSS Inc., Chicago, IL, USA). Non-parametric data were expressed as median with interquartile range and parametric data as mean with standard deviation. Baseline demographic and clinical characteristics were compared using χ^2^ test for categorical variables, Kruskal–Wallis test for non-parametric continuous variables and one-way ANOVA for parametric continuous variables. *P* values of < 0.05 were considered statistically significant. Univariate binary logistic regression analysis was performed to assess whether BMI affected conversion to open or morbidity and mortality, with morbidity and mortality defined as the presence of any of the following outcomes: 30-day reoperation, 30-day readmission, anastomotic leak and 30-day mortality. Following this, a multivariate model was applied were BMI was adjusted for all clinically relevant variables. Finally, a univariate and multivariate linear regression model was applied to investigate the effect of BMI on operative time. Since both BMI and operation time were non-parametric, Spearman’s rank correlation was calculated to evaluate the correlation.

## Results

A total of 1386 patients underwent minimally invasive colorectal resection surgery by a surgeon from each study site. Of those, 84 (6%) were morbidly obese, 246 (18%) obese and 1056 (76%) non-obese.

### Baseline characteristics

The baseline characteristics of the three groups are summarised in Table 1. Patient baseline characteristics were similar for age, diagnosis, operating surgeon, surgical approach and T stage but differed in terms of ASA grade, gender and mode of surgery.

**Table 1:** Baseline characteristics

	Non-obese (*n* = 1056)	Obese (*n *= 246)	Morbidly obese (*n* = 84)	*p* value
Median BMI	25 (22.7–27)	31 (30–32)	36 (35.1–39)	**0.000** ^**a**^
Median age	68 (58–77)	65 (58.2–73.9)	65 (58-72.2)	0.056^a^
Gender				
Male	563 (53.3%)	151 (61.4%)	35 (41.7%)	**0.005** ^b^
Female	493 (46.7%)	95 (38.6%)	49 (58.3%)	
ASA grade				
I	159 (15.4%)	27 (11.1%)	3 (3.7%)	**0.000** ^b^
II	680 (65.8%)	164 (67.5%)	41 (50%)	
III	191 (18.5%)	51 (21%)	37 (45.1%)	
IV	4 (0.4%)	1 (0.4%)	1 (1.2%)	
Diagnosis				
Malignant	784 (74.2%)	193 (78.5%)	66 (78.6%)	0.297^b^
Benign	272 (25.8%)	53 (21.5%)	18 (21.4%)	
Mode of surgery				
Elective	1015 (96.1%)	244 (99.2%)	82 (97.6%)	**0.045** ^b^
Emergency	41 (3.9%)	2 (0.8%)	2 (2.4%)	
Surgical approach				
Laparoscopic	979 (92.7%)	226 (91.9%)	81 (96.4%)	0.371^b^
Robotic	77 (7.3%)	20 (8.1%)	3 (3.6%)	
Operating Surgeon				
A	778 (73.7%)	181 (73.6%)	64 (76.2%)	0.927^b^
B	30 (2.8%)	7 (2.8%)	1 (1.2%)	
C	248 (23.5%)	58 (23.6%)	19 (22.6%)	
T stage				
0	29 (3.8%)	9 (4.7%)	2 (3%)	0.116^b^
1	70 (9.1%)	24 (12.6%)	6 (9%)	
2	170 (22.1%)	43 (22.6%)	24 (35.8%)	
3	397 (51.6%)	95 (50%)	32 (47.8%)	
4	104 (13.5%)	19 (10%)	3 (4.5%)	

Statistically significant values are given in bold

^a^Kruskal–Wallis Test

^b^Chi-square

As expected ASA grade was worse in the morbidly obese group. There were more female patients in the morbidly obese group and less in the obese group compared to the non-obese group (female patients in morbidly obese vs obese vs non-obese: 58.3% vs 38.6% vs 46.7%; *p* = 0.005). The obese group had the least amount of patients receiving emergency surgery and the non-obese group the most (morbidly obese vs obese vs non-obese: 2.4% vs 0.8% vs 3.9%; *p* = 0.045). The operative procedures performed across the 3 groups are summarised in Table 2.

**Table 2:** Operative procedures

	Non-obese (*n* = 1056)	Obese (*n* = 246)	Morbidly obese (*n* = 84)
Right hemicolectomy	280 (26.5%)	41 (16.7%)	16 (19%)
Extended right hemicolectomy	48 (4.5%)	8 (3.3%)	3 (3.6%)
Left hemicolectomy	24 (2.3%)	6 (2.4%)	4 (4.8%)
Sigmoid colectomy	34 (3.2%)	16 (6.5%)	4 (4.8%)
Anterior resection	467 (44.2%)	136 (55.3%)	40 (47.6%)
Abdominoperineal excision	41 (3.9%)	11 (4.5%)	10 (11.9%)
Hartman’s procedure	21 (2%)	4 (1.6%)	2 (2.4%)
other	141 (13.4%)	24 (9.8%)	5 (6%)

### Perioperative characteristics and outcomes

The perioperative characteristics of the three groups are summarised in Table 3. Operation time and blood loss were different across the three cohorts (morbidly obese vs obese vs non-obese: median operation time 185 vs 188 vs 170 min, *p* = 0.000; median estimated blood loss 20 vs 20 vs 10 ml, *p* = 0.003). Conversion rate was similar across the three groups with an overall conversion rate of 1.2% (*p* = 0.251).

**Table 3:** Perioperative characteristics and outcomes

	Non-obese (*n* = 1056)	Obese (*n* = 246)	Morbidly obese (*n* = 84)	*p* value
Median operative time (min)	170 (125–210)	188 (145–240)	185 (145–210)	**0.000** ^a^
Median estimated blood loss (ml)	10 (0–20)	20 (0–45)	20 (10–50)	**0.003** ^a^
Conversion to open	11 (1%)	5 (2%)	0	0.251^b^

Statistically significant values are given in bold

^a^Kruskal–Wallis Test

^b^Chi-square

### Post-operative clinical and pathological outcomes

There were no differences in any of the post-operative clinical outcomes (length of stay, 30-day readmission rate, 30-day reoperation rate, anastomotic leak rate, 30-day mortality rate) or pathological outcomes (lymph node yield and CRM clearance) between the three cohorts as summarised in Table 4.

**Table 4:** Post-operative clinical and pathological outcomes

	Non-obese (*n* = 1056)	Obese (*n* = 246)	Morbidly obese (*n* = 84)	*p* value
Median length of stay (days)	4 (3–7)	5 (3–7)	4 (3–7)	0.454^a^
30-day readmission	115 (10.9%)	28 (11.4%)	14 (16.7%)	0.274^b^
30-day reoperation	31 (2.9%)	5 (2%)	4 (4.8%)	0.427^b^
30-day mortality	5 (0.5%)	0	0	0.454^b^
Anastomotic leak	14 (1.5%)	3 (1.3%)	2 (2.8%)	0.663^b^
Median lymph node yield	16.5 (12–23)	16 (11–23)	18 (14–22.75)	0.267^a^
R0 clearance	743 (94.8%)	190 (98.4%)	66 (100%)	0.079^b^

^a^Kruskal–Wallis Test

^c^Chi-square

### Logistic and linear regression analysis

Univariate logistic regression analysis showed that BMI did not affect conversion to open for the participants in this study. This was still the case in multivariate analysis when other clinically relevant factors were adjusted for (age, ASA grade, mode of surgery, diagnosis). Findings are summarised in Table 5. Furthermore, univariate logistic regression analysis (Table 6) showed that BMI did not affect morbidity and mortality. This was still the case in multivariate analysis when other clinically relevant factors were adjusted for (age, ASA grade, mode of surgery, diagnosis).

**Table 5:** Univariate and multivariate logistic regression for conversion

	Univariate			Multivariate		
	OR	95% CI	*p* value	OR	95% CI	*p* value
BMI	1.016	0.927–1.114	0.731	1.029	0.935–1.133	0.556
Age	1.006	0.973–1.040	0.714	1.023	0.985–1.063	0.241
ASA grade	0.965	0.422–2.205	0.933	0.844	0.357–1.996	0.700
Mode of surgery (emergency)	0.498	0.064–3.852	0.504	0.483	0.059–3.916	0.495
Diagnosis (malignant)	1.839	0.663–5.098	0.241	2.592	0.818–8.211	0.105

*OR* odds ratio, *CI* confidence interval

**Table 6:** Univariate and multivariate logistic regression for morbidity and mortality

	Univariate			Multivariate		
	OR	95% CI	*p* value	OR	95% CI	*p* value
BMI	1.011	0.981–1.041	0.492	1.005	0.975–1.037	0.738
Age	0.994	0.985–1.004	0.233	0.994	0.982–1.007	0.356
ASA grade	1.222	0.938 – 1.590	0.137	1.298	0.975–1.728	0.074
Mode of surgery (emergency)	0.719	0.330–1.569	0.408	0.777	0.349–1.732	0.537
Diagnosis (malignant)	1.302	0.925–1.302	0.130	1.197	0.789–1.815	0.398

*OR* odds ratio, *CI* confidence interval

**Fig. 1:**
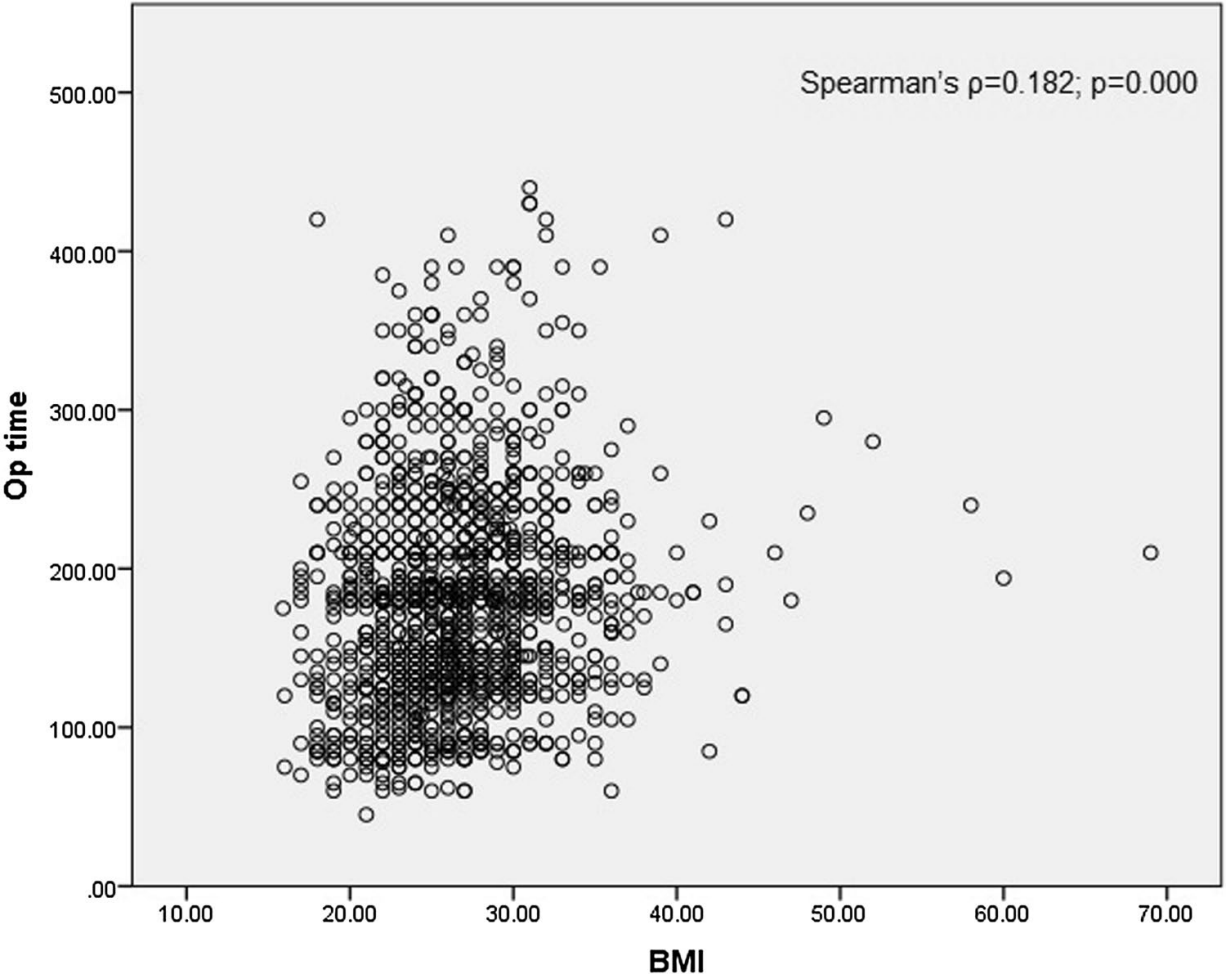
Scatter plot of BMI against operative time

There was however, a weak but significant correlation between BMI and operative time (Spearman’s *ρ* = 0.182; *p* = 0.000). Univariate linear regression analysis showed that for every one-unit increase in BMI operative time increases by roughly 2 min (*b* = 2.243, 95% CI 1.524–2.962; * p*= 0.000). This was still the case in multivariate analysis when other clinically relevant factors were considered (ASA grade, mode of surgery) (*b* = 2.295, 95% CI 1.554–3.036; *p* = 0.000). Findings are summarised in Table 7. Figure 1 represents the scatter plot of BMI against operative time.

**Table 7:** Univariate and multivariate linear regression for operative time

	Univariate			Multivariate		
	Estimate (beta)	95% CI	*p* value	Estimate (beta)	95% CI	*p* value
BMI	2.243	1.524 to 2.962	**0.000**	2.295	1.554 to 3.036	**0.000**
ASA grade	− 4.220	− 10.467 to 2.027	0.185	− 6.323	− 12.540 to − 0.106	**0.046**
Mode of surgery (emergency)	− 17.671	− 38.817 to 3.474	0.101	− 12.939	− 34.269 to 8.390	0.234

Statistically significant values are given in bold

*CI* confidence interval

Correlation for BMI: Spearman’s *ρ* = 0.182; *p* = 0.000

### Subgroup analyses

#### Patients with 35 ≤ BMI < 40 vs BMI ≥ 40

There were 64 patients with a BMI between 35 and 40 and 20 with a BMI equal or greater than 40. Patients with a BMI ≥ 40 were younger (66 vs 59.5; *p* = 0.044). There were no other significant differences in any of the baseline characteristics or short-term surgical outcomes between the two groups. Operation time appeared to be higher in the BMI ≥ 40 group but this was not statistically significant (180 vs 194 min; *p* = 0.142).

#### Right colonic, left colonic and rectal resections

There was a total of 396 patients that received right colon, 88 left colon and 732 rectal resections. The remaining patients received a combination of subtotal colectomies, proctocolectomies and panproctocolectomies.

For the right colonic resections, the results were similar to those of the overall cohort, although operation time and blood loss did not reach statistical significance (morbidly obese vs obese vs non-obese: median operation time 125 vs 125 vs 115 min, *p* = 0.101; median estimated blood loss 20 vs 20 vs 10 ml, *p* = 0.065). There were no observed differences in terms of baseline characteristics or short-term surgical outcomes between the three cohorts in left colonic resections.

In rectal resections, results reflected again those of the overall cohort results (ASA higher with increasing BMI, operation time and blood loss higher in obese and morbidly obese groups) and there were no differences in any of the short-term post-operative outcomes between the three groups with the exception of readmission rate, which was higher in the morbidly obese group (non-obese vs obese vs morbidly obese: 10.2, 9.9, and 23.1%; *p* = 0.016).

## Discussion

Morbid obesity is becoming increasingly more common and the surgical outcomes of this group of patients warrant further investigation [15, 16]. In this study, we have found that the increased technical difficulty encountered in obese and morbidly obese patients in minimally invasive colorectal surgery results in higher operative times and blood loss. However, conversion rate, length of stay, 30-day readmission, 30-day reoperation, anastomotic leak and 30-day mortality rates were similar between non-obese, obese and morbidly obese patients. In addition, our results demonstrate that in cancer patients there were no differences in lymph node yield and CRM (R0) clearance rates between the three cohorts. Furthermore, BMI was not found to affect conversion rate or morbidity and mortality on logistic regression analysis. These findings strengthen the argument that by standardising operative technique and post-operative care minimally invasive surgery for the morbidly obese and obese is safe, feasible and does not result in higher surgical morbidity.

In our study we found that operative time and estimated blood loss were higher in the obese and morbidly obese groups. However, the operative time and blood loss of the obese and morbidly obese groups were similar. This is different to what we anticipated, since the increasing technical difficulty associated with operating on the morbidly obese patients was expected to result in even longer operative times and higher blood loss. Furthermore, it should be noted that although statistically significant, the differences in operative time and blood loss are not clinically significant. Patients in the obese and morbidly obese groups took an extra 15 min to operate on and had an additional 10 mls of blood loss. Considering the median operative time for the non-obese was over 3 h, the longer operative time and higher blood loss encountered in the obese and morbidly obese are unlikely to significantly affect the patient’s clinical outcomes as our results infers.

Our results are in accordance with a recently published meta-analysis [10] examining the outcomes of obese vs non-obese laparoscopic colorectal surgery patients. This study combined the data of 13 and 6 studies for operative time and blood loss respectively and found that both parameters were higher in the obese group, by an average of 13 min and 34 mls, respectively. The operative time and blood loss of obese vs non-obese patients receiving laparoscopic colorectal surgery was also examined in a recently published systematic review including 30 studies [13]. In this review it is clear that there is great variability in reported outcomes. Regarding operative time 18 studies reported longer operative times in the obese vs 12 that did not. In terms of estimated blood loss, 8 studies reported higher blood loss in the obese and 8 found no difference between the two groups. The differences in reported outcomes are probably multifactorial. First of all, studies failing to demonstrate a difference could be doing so due to a type 2 error. This is because the actual differences are small and therefore a large sample size is required to demonstrate a statistically significant difference in outcomes. A second reason might be variability in surgical practise. The operative technique and experience of some surgeons might be better adapted to cope with the increased technical difficulties of obese patients, therefore making less likely to demonstrate any differences in surgical outcomes.

In our study, we found that BMI was as independent factor for operative time as demonstrated in linear regression analysis. Although the actual correlation was weak (*ρ* = 0.182), it was statistically significant and linear regression analysis showed that for every increase in one unit of BMI operative time increased by roughly 2 min. We are not the first study to demonstrate that BMI is an independent predicting factor for operative time, with three previously published studies demonstrating similar results [22–24], one of which compared the operative times of morbidly obese (BMI ≥ 35) patients to non-obese patients [22].

Apart from operative time and blood loss, there were no statistically significant differences in short-term surgical outcomes between the three groups. This is opposite to what one might expect, considering again the increased technical difficulties encountered when operating on obese and even more so on morbidly obese patients. Multiple previous studies (including systematic reviews and meta-analysis) have shown that obesity is associated with a higher conversion rate, anastomotic leak rate, increased post-operative morbidity and a lower lymph node yield in minimally invasive surgery [10–12, 25]. However, several studies have reported similar short-term surgical outcomes between obese and non-obese patients [9, 13, 14]. Just as for operative time and blood loss, the reasons behind this are probably multifactorial. Variability in surgical practice, surgeon experience and centre volume can all effect surgical outcomes. Furthermore, small sample sizes and small outcome differences reduce the prospect of demonstrating a statistically significant difference. We believe that by standardising operative technique and breaking down surgical procedures in digestible modules we enhance reproducibility of results and facilitate surgical training. This way, surgical outcomes are fairly similar, regardless of perioperative conditions. This could account for the similar short-term surgical outcomes in our patients, regardless of their obesity level.

Only a handful of studies have specifically examined the morbidly obese [14, 25–27], most of them using a cut-off of BMI ≥ 40 [14, 25, 26]. Hussan et al. examined 85,300 discharges of colorectal cancer surgery patients from the US 2012 National Inpatient Sample and found that morbid obesity was associated with a higher prevalence of perioperative comorbidities, surgical complications, conversions to open, perioperative mortality and prolonged length of stay [25]. However, it should be noted that in this study 70% of patients received open surgery. In contrast, Khoury et al. [14] specifically examined the feasibility of laparoscopic surgery in the morbidly obese. In this study, there was no statistical difference in any surgical outcomes between the morbidly obese and non-obese (36 vs 36 patients) apart from skin incision length. However, the authors have reported a trend towards worse short-term outcomes in the morbidly obese group. This could be suggested with some of our results. Readmission, reoperation and anastomotic leak rates appear higher in the morbidly obese group when compared to the obese and non-obese groups (Table 4). Nonetheless, these differences are small and not statistically significant. In addition, when examining whether BMI affected conversion rate or morbidity and mortality in a logistic regression model no association was demonstrated.

A recently published American study by Champagne et al. examined the outcomes of obese patients having laparoscopic colectomies based on the degree of obesity [26]. In this study, obese patients were divided in three groups (obese, morbidly obese and super-obese) and had their outcomes evaluated. This manuscript concluded that increasing obesity severity correlated with worse perioperative outcomes. However, the majority of the short-term outcomes presented (operative time, conversion rate, post-operative morbidity and length of stay) are similar between the obese and morbidly obese groups, with these outcomes only worsening in the super-obese group (BMI ≥ 50). In terms of the morbidly obese, their results are similar to our findings, demonstrating no real differences between morbidly obese and obese patients. The worse short-term outcomes presented in the super-obese group could be secondary to the greatly increased visceral adiposity and abdominal wall size, further adding to the technical complexity of the operation. In our study population evaluation of the super-obese surgical outcomes was prohibited due to the small sample size (*n* = 4).

Subgroup analysis of the data according to right colon, left colon and rectal resections widely demonstrated similar results to those of the overall cohort. However, it should be noted that readmission rate was higher in the morbidly obese group for rectal resections, while similar between the obese and non-obese groups. It might be that in patients with morbid obesity (BMI ≥ 35) rectal resection presents a technical challenge of even tighter pelvic space occupied by a rather large mesorectum which may result in even greater intra-operative difficulty, leading to higher morbidity reflected by a higher readmission rate. However, this is an isolated positive finding that was not reconfirmed in logistic regression analysis (data not shown).

The main strengths of this study are its large sample size, the fact that data were collected from three centres from two different countries and the fact that all three participating surgeons follow the same modular standardised operative techniques. However, as with all research there are specific limitations that should be duly recognised and acknowledged. Despite collecting data from prospectively collated databases, the study design was retrospective in nature. However, collecting data from prospectively maintained databases minimises observation bias and by including all consecutive patients’ selection bias is minimised. Secondly, we have defined morbid obesity as BMI ≥ 35 in accordance with the NIH conference [17]. However, many studies examining morbid obesity use BMI 40 as a cut-off. Although a BMI ≥ 40 would better allow us to evaluate the effect of an ever-increasing BMI on surgical outcomes, we only had a small number of patients with BMI ≥ 40 (*n* = 20). A subgroup analysis of patients with 35 ≤ BMI < 40 vs BMI ≥ 40 showed no significant differences between the two groups, but results should be taken with caution due to the small sample size. A further limitation in our study is that there are differences in the baseline characteristics in terms of ASA grade, mode of surgery and gender between the three examined cohorts. ASA grade increased across the obesity categories as one would expect, but the differences in gender and mode of surgery appear random. Nevertheless, the differences in gender and mode of surgery are relatively small. Moreover, ASA grade and mode of surgery have been accounted for in the multivariate logistic regression analysis.

In summary, our study’s findings show that minimally invasive colorectal surgery is safe and feasible regardless of BMI when operative technique and perioperative care are standardised. The increased operative time and blood loss observed in the obese and morbidly obese are of no clinical significance and have not been proved to affect the rest of the short-term surgical outcomes. Therefore, this group of patients is likely to benefit from the advantages offered by minimally invasive surgery. Larger scale multi-centre observational studies are required to determine the outcomes of morbidly obese patients receiving minimally invasive colorectal surgery.
